# UniPep - a database for human *N*-linked glycosites: a resource for biomarker discovery

**DOI:** 10.1186/gb-2006-7-8-r73

**Published:** 2006-08-10

**Authors:** Hui Zhang, Paul Loriaux, Jimmy Eng, David Campbell, Andrew Keller, Pat Moss, Richard Bonneau, Ning Zhang, Yong Zhou, Bernd Wollscheid, Kelly Cooke, Eugene C Yi, Hookeun Lee, Elaine R Peskind, Jing Zhang, Richard D Smith, Ruedi Aebersold

**Affiliations:** 1 Institute for Systems Biology, Seattle, WA 98103, USA; 2 NYU Center for Comparative Functional Genomics, New York, NY, USA; 3 Institute for Molecular Systems Biology, ETH Zurich and Faculty of Sciences, University of Zurich, Switzerland; 4 VA Puget Sound Health Care System, Seattle, WA 98108, USA; 5 Harborview Medical Center, University of Washington School of Medicine, Seattle, WA 98104, USA; 6 Biological Sciences Division and Environmental Molecular Sciences Laboratory, Pacific Northwest National Laboratory, Richland, WA 99352, USA

## Abstract

There has been considerable recent interest in proteomic analyses of plasma for the purpose of discovering biomarkers. Profiling *N*-linked glycopeptides is a particularly promising method because the population of *N*-linked glycosites represents the proteomes of plasma, the cell surface, and secreted proteins at very low redundancy and provides a compelling link between the tissue and plasma proteomes. Here, we describe UniPep  - a database of human *N*-linked glycosites - as a resource for biomarker discovery.

## Abstract 2

UniPep, a database of human N-linked glycosites is presented as a resource for biomarker discovery

## Rationale

It is generally understood that variations in an individual's genetic background and physiologic state give rise to alterations in the person's plasma protein profile. (For the purposes of this report, the terms 'serum' and 'plasma' are used interchangeably.) Of particular interest are those changes that reflect important processes in specific organs or tissues, such as the early onset of pathologic processes or the response to pharmacologic intervention. The detection and correct interpretation of the respective plasma proteome patterns are expected to realize a significant benefit for human health through the development of simple blood tests for (early) detection and stratification of many of the common serious human diseases (for example, cancers, neurodegenerative disorders, and diabetes, among others). The great potential impact of the information contained in the plasma proteome has resulted in a strong focus of applying a range of proteomic strategies to discover and detect relevant plasma proteome markers or patterns [1-7].

Several factors complicate plasma proteomic analyses in general and specifically the detection of proteins in plasma that are derived from a particular tissue. Complications include the enormous complexity of the plasma proteome, the high dynamic range of protein concentrations, the dominance of the plasma proteome by few highly expressed proteins, and the expected substantial dilution of tissue-derived proteins in the large pool of an individual's blood [8]. In addition, it appears that the plasma protein composition varies substantially between individuals in a population [9] and within an individual as a function of a multitude of factors, including sex, age, general health, and external and lifestyle influences [10,11]. Partly as a result of these complications, attempts to discover sensitive and selective biomarkers using the available proteomic strategies, including two-dimensional gel electrophoresis [3], shotgun tandem mass spectrometry (MS/MS) [1,2,7,12,13], surface-enhanced laser/desorption ionization (SELDI)-MS [14], and others, have met with modest success. In fact, at this point not a single validated biomarker has been identified using these proteomic methods. Careful analysis of the results produced by such studies has indicated the restricted dynamic range of the analytical methods used as a main limitation [15]. Each one of the methods has demonstrated ability to reliably detect and identify quantitative changes in proteins in the top two to four orders of magnitude of the dynamic range of the plasma proteome, which is thought to span minimally 10 orders of magnitude. Therefore, current methods are largely blind to the majority of plasma proteins, especially to those that are released by specific tissues at low concentrations.

The current most promising strategy to overcome these limitations is to fractionate the plasma proteome into minimally overlapping fractions and to analyze by MS each fraction separately. In addition to fractionation schemata based on physicochemical properties of proteins and peptides such as size, charge, and hydropathicity, the specific selection of subproteomes that contain a particular functional group and the depletion of plasma for highly expressed proteins have been successfully applied [16].

Our group introduced a method for the selective isolation of *N*-linked glycopeptides, and analysis of the complex peptide mixture representing the now de-glycosylated forms of these peptides by MS/MS [17]. This method further enables high-throughput identification of *N*-linked glycosylation sites (*N*-linked glycosites), defined as the acceptor asparagines for *N*-linked glycosylation to take place on protein sequences. By selectively isolating this subset of peptides, the procedure achieves a significant reduction in analyte complexity at two levels. First, it reduces the total number of peptides because of the fact that every plasma protein on average only contains a few *N*-linked glycosites. Second, it reduces pattern complexity by removing the oligosaccharides that contribute significantly to the peptide pattern heterogeneity. We have shown that application of the method to plasma results in a significant reduction in sample complexity, increased sample throughput, and increased dynamic range for proteome analysis [17,18]. However, the most significant benefits from the selective analysis of *N*-linked glycopeptides originate from the fact that the number of *N*-linked glycosites in the human proteome is modest, known in principle, and identifiable with current technology.

This situation has profound conceptual and experimental implications for biomarker discovery. First, biomarker discovery research using this approach operates in a defined space; all of the biomarkers discovered by the method for any disease will be a subset of the known *N*-linked glycosites. The benefits of navigating in a mapped space as opposed to *de novo *discovery of the observable events in each experiment have been impressively demonstrated by the genomic sciences. Second, the data units generated by the method are specific *N*-linked glycosites; therefore comparison between studies, labs and disease types is significantly simplified. It will, for instance, become trivial to compare a biomarker data set for a particular disease with the one generated for different diseases to determine whether the putative marker is disease specific or a pan-disease marker. Third, the relatively modest number of possible *N*-linked glycosites will facilitate the development of targeted approaches for high-throughput proteomic screening, for instance via screening ordered peptide arrays by matrix-assisted laser desorption/ionization (MALDI)-MS/MS [19,20]. Finally, the same pool of *N*-linked glycosites can be explored to generate potential marker patterns from the cell surface and secreted protein populations of cells and tissues, and for the targeted search for such tissue-derived patterns in plasma, thus dramatically reducing the challenge of defining biomarker patterns from global plasma protein profiles. It is therefore apparent that knowledge of all *N*-linked glycosites of the human proteome and their organization in a relational database would be of significant interest for protein biomarker discovery.

In this report we describe UniPep, which is a database for human *N*-linked glycosites that can be interrogated via the internet [21]; the informatics infrastructure to populate the database with data of consistent quality; and an initial set of 1522 unique *N*-linked glycosites identified at high confidence, representing an estimated 3% of the total number of *N*-linked glycosites of the human proteome and 7% of the *N*-linked glycosites from proteins predicted as being secreted or transmembrane proteins.

## Results and discussion

### UniPep: a database for human N-linked glycosites

*N*-linked glycosites generally fall into the N-X-S/T sequence motif, in which X denotes any amino acid except proline [22]. The number and distribution of the *N*-linked glycosites over the human proteome can therefore be computationally determined by scanning the sequences for the presence of the motif. To display all the theoretical *N*-linked glycosites in the human International Protein Index (IPI) database (version 2.28) and to relate them to the *N*-linked glycosites that were experimentally observed by mass spectrometric analysis, we developed the UniPep database and web interface [21]. The potential *N*-linked glycosites were parsed and loaded into a relational database and the data are easily searchable using SQL (structured query language). User access to the database is provided via a cgi web interface, which is part of the larger application framework named Systems Biology Experiment Analysis Management System relational database (SBEAMS [23]).

The primary user interface is a search page that allows users to search the data based on various parameters and supports the use of wild card characters. Possible search parameters include amino acid sequence, gene symbol, gene name, Swiss-Prot accession number, or IPI accession number. When a search is executed a list of all proteins that match the search criteria is shown. Each listing contains a link to view a detailed record for the respective protein.

**Figure 1:**
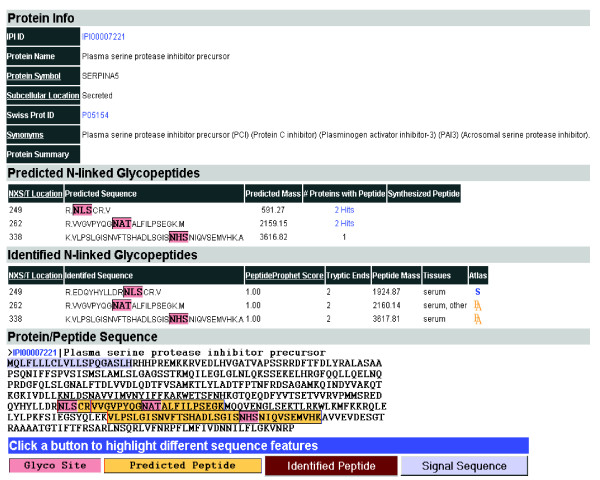
Representative output of *N*-linked glycosites from database using UniPep. UniPep contains all proteins in the International Protein Index (IPI) database (version 2.28) with at least one *N*-linked glycosite and allows users to view all the predicted and identified *N*-linked glycosites from a specific protein. For each potential *N*-linked glycoprotein, a user can see the protein annotation, predicted subcellular location, and sequence(s) of predicted *N*-linked glycosites(s). The uniqueness of a peptide in the database is also presented as number of hits in the database, and for those peptides present in multiple proteins, linkage to other proteins in the database is provided. If any predicted *N*-linked glycosite was identified in the dataset from this study, then it is listed as an identified peptide with PeptideProphet score [39] to allow researchers to evaluate the confidence of the identification. The sequence of the proteins queried is overlaid with different sequence features such as the *N*-linked glycosites, the predicted and identified peptide sequences, signal peptide, and transmembrane segment(s) [21].

For each protein in the UniPep database, we display four different types of information (Figure 1). The first section, Protein Info, indicates the predicted subcellular location of the protein along with other information about the respective protein from Entrez Gene [24]. *N*-linked glycosylation is enriched in proteins destined for extracellular environments [25]. These include proteins on the extracellular side of the plasma membrane (cell surface proteins), transmembrane proteins, and secreted proteins. We predicted the subcellular localization of each protein based on whether a protein contains a signal peptide (computed using the program SignalP 2.0 [26]) and/or transmembrane region(s) (computed using the program TMHMM [version 2.0] [27]). The proteins were thus categorized as cell surface, secreted, transmembrane, or intracellular.

In the second section, Predicted *N*-linked Glycopeptides, the sequences of potential tryptic *N*-linked glycosites and their location within the protein sequence are displayed. Some potential *N*-linked glycopeptides (7.9% of unique *N*-linked glycopeptides) contain multiple N-X-S/T sites within a predicted tryptic peptide; in this case, each N-X-S/T site was considered an *N*-linked glycosite. We also determined the uniqueness of each predicted *N*-linked glycosite by searching the entire IPI protein database for the number of occurrences of the respective sequence in different proteins. The results of these analyses are annotated under 'number of proteins with peptide' (Figure 1).

In the third section, Identified *N*-linked Glycopeptides, the mass spectrometrically identified peptides along with relevant annotations are displayed. For the identified *N*-linked glycosites, sequences from SEQUEST search result were mapped to the potential *N*-linked glycosites from the IPI database and the overlapping sequences containing the same *N*-linked glycosites were resolved to generate nonredundant *N*-linked glycopeptide (see rules below). For the protein in Figure 1 all of the predicted *N*-linked glycosites were indeed observed, although the site at position 249 was observed as a peptide with a missed tryptic cleavage site immediately preceding the site of carbohydrate attachment.

In the fourth section, Protein/Peptide Sequence, the whole protein sequence is indicated and the signal peptides, transmembrane sequences, and identified *N*-linked glycosites are highlighted to give a general indication of protein topology.

Table 1 details the number of predicted unique *N*-linked glycosites in the human proteome and their distribution over the cell surface, secreted, transmembrane, or intracellular fractions. The table also indicates the degree of simplification achieved by focusing on the *N*-linked glycosites compared with analysis of the whole proteome, assuming occupancy of each potential *N*-linked glycosite.

Without considering possible sequence variation and post-translational modifications of each peptide, 749,163 unique tryptic peptides within a mass range of 500-5000 are expected from the protein entries in the IPI database. Of these, 52,442 unique peptides (7.0%) contain potential *N*-linked glycosites. These 7.0% N-X-T/S containing peptides represent 67.5% of the proteins in the database. Furthermore, only about 33.4% of proteins (13,389 protein entries) from the human protein database are predicted to be exposed to an extracellular environment and therefore are likely to be glycosylated [28]. These predicted extracellular proteins contain 22,692 unique N-X-T/S motif containing peptides representing 3.0% of the total unique tryptic peptides. These 3.0% of peptides represent 9583 protein entries (71.6% of 13,389 proteins predicted as being extracellular proteins; Table 1). This suggests that the number of *N*-linked glycosites in the human proteome is modest (3.0% of total expected peptides), known in principle, and identifiable with current technology. *N*-linked glycopeptide analysis therefore targets a relatively small fraction of peptides from complex human plasma proteome that are enriched for the proteins exposed to extracellular side of the plasma membrane. The modest number of potential *N*-linked glycosites indicates that the selective isolation of these peptides results in a substantial reduction in the redundancy inherent in serum proteome analysis and that the concentration limit of detection is therefore significantly improved because of the reduction in sample complexity [18].

Analysis of *N*-linked glycosites reveals potential biomarkers that change in glycoproteins and glycosite occupancy; this is supported by the observation that most known clinical protein markers are also known to be glycosylated. The reduction in sample complexity is beneficial for achieving higher sensitivity for low abundance proteins, but it also leads to the loss of some, potentially important information. Potential disease markers that are due to changes in nonglycosylated proteins, other protein post-translational modifications, and oligosaccharide structures will not be detected at a glycopeptide level.

### Informatics infrastructure for automatic and consistent data processing in UniPep

The utility of the UniPep database as a public resource depends on the number of *N*-linked glycosites identified by MS at high confidence. The limited number of *N*-linked glycosites in the human proteome suggests that all or at least the majority of these peptides can be identified if respective data from different experiments and laboratories are integrated into a single comprehensive database. We therefore developed an informatics infrastructure for the identification of *N*-linked glycosites from MS/MS spectra at consistent process, irrespective of the origin of the raw data. The system builds on SBEAMS [23] and the tools, procedures, and statistical models developed for the PeptideAtlas project [29-31] and the Trans Proteomic Pipeline (TPP) [32].

**Figure 2:**
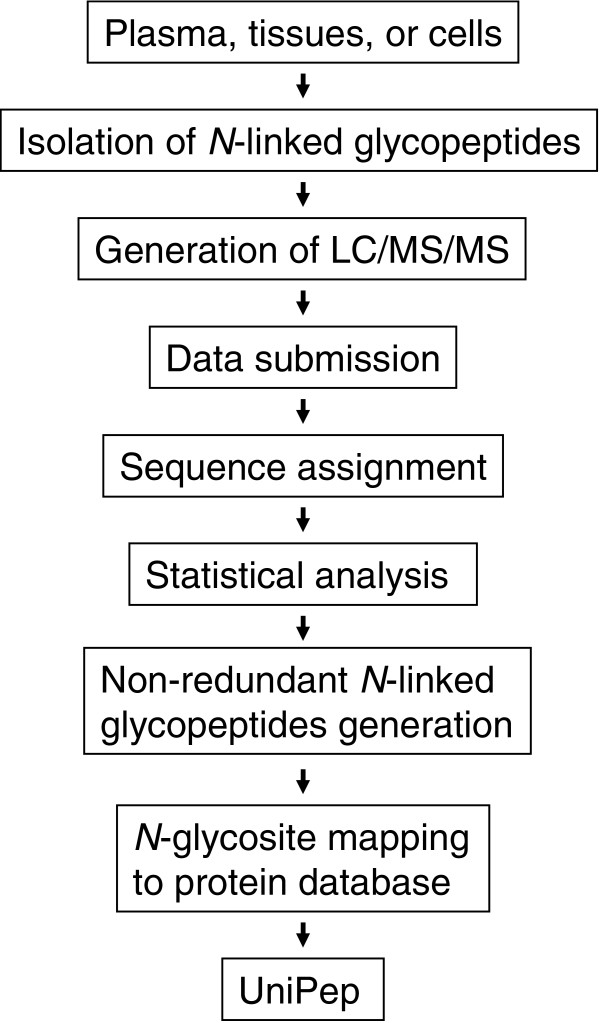
Consistent analysis pipeline. Shown is a schematic presentation of consistent analysis pipeline for the identification of high-quality *N*-linked glycosites using glycopeptide capture and LC-MS/MS. LC, liquid chromatography; MS/MS, tandem mass spectrometry.

The procedure to add new data to UniPep consists of the following five steps (Figure 2). In step 1, data submission, raw MS/MS data from any type of tandem mass spectrometer can be submitted and processed. The spectra are formatted, preferably into mzXML [33] or mzData (HUPO Proteomics Standards Initiative), which are open file formats for the representation of MS data. Other data formats will be translated into these formats and are therefore also acceptable.

In step 2, sequence assignment, the MS/MS data are searched against a database (IPI version 2.28 for the current version of UniPep) by SEQUEST to correlate MS/MS spectra with the amino acid sequences of the peptides. Other database search engines, such as COMET [32], MASCOT [34], and ProbID [35], can also be used because they are supported by current TPP [32] and UniPep. Support for several other search engines, such as X!Tandem [36], PHENYX [37], and OMSSA [38] is planned in subsequent TPP releases, and would thus be supported by UniPep.

Statistical analysis, step 3, involves further analysis of assigned peptide sequences using PeptideProphet [39]. Based on the distribution of scores over the whole dataset, PeptideProphet calculates for each peptide a probability of the assignment being correct. The information used by PeptideProphet includes database search scores, difference between the measured and theoretical peptide mass, the number of termini consistent with the type of enzymatic cleavage used, the number of missed cleavage sites, and other factors. PeptideProphet also calculates for each dataset false-positive and false-negative error rates at specific probability score cutoff values [40]. A minimum PeptideProphet probability score of ≥0.5 was initially used to remove low probability peptides. Using a probability score of ≥0.5 as the cutoff, the estimated false-positive and false-negative rates generally fall below 10% and 20%, respectively (Table 2). The identified peptide sequences with their probability score and the corresponding MS/MS spectra are output using INTERACT for inclusion in the database [41].

In step 4, nonredundant *N*-linked glycopeptide generation, peptides with overlapping sequences containing the same (for example, redundant) N-X-S/T sequons from the same dataset are resolved in favor of those sequences that contain the greater number of tryptic ends, a lower number of miss-cleaved internal tryptic sites, and higher PeptideProphet probability. The fifth and final step is *N*-linked glycosite mapping to protein database. The peptide sequences from the nonredundant list constitute sequence patterns that are used to match each peptide against the corresponding *N*-linked glycosite in the IPI database. This step results in a set of IPI numbers with the location of each specific N-X-T/S site to which the given peptide will match. These locations are concatenated into a unique key (for instance, IPI00000001 site 327 becomes IPI00000001.327), and occurrence of the matching peptide object is mapped to each key within *N*-linked glycosites in UniPep. If a peptide has already been mapped to a particular IPI.N-X-T/S key, then the new and existing peptides are merged (as described in step 4, described above) and the better peptide is chosen.

This procedure ensures the highest degree of consistency for data in UniPep. All MS/MS spectra are stored and available in the mzXML files in the SBEAMS - Proteomics database [23], from which UniPep is derived. Thus, collectively, the steps in this procedure produce a database, UniPep, that contains a minimal set of peptides containing the consensus *N*-linked glycosylation motif, the MS/MS spectra representing the peptide, and the likelihood that the peptide has been correctly identified (Figure 1).

Only peptides containing consensus *N*-linked glycosites (the N-X-T/S motif) are used to predict the potential *N*-linked glycosites from protein sequences in the database, and only the identified peptides containing the *N*-linked glycosites are used to map to the potential *N*-linked glycosites. Peptides not containing the sequon can come from three sources. The first is from peptides resulting from nonspecific isolation in the glycopeptide isolation procedure, the second from incorrect peptide sequence assignments (false positives), and the third from atypical *N*-linked glycosylation in which glycosylation occurs in sequences other than the consensus N-X-S/T motif such as N-X-C motif [42]. Currently, we exclude atypical *N*-linked glycopeptides in UniPep database because of lack of understanding of consensus atypical sequence motifs. Peptides not containing N-X-S/T motif were stored in PeptideAtlas [29,43], and peptide identification information including sequence, PeptideProphet, and number of times each sequence was identified was recorded and displayed in PeptideAtlas. A link to PeptideAtlas is provided for each identified peptide and protein in the column entitled 'Atlas'. This provides a number of links to other resources, such as ENSEMBLE, via PeptideAtlas (Figure 1).

It is understood that nearly all large-scale datasets obtained using high-throughput methods contain a certain fraction of false-positive data. Thus, estimation of false-positive error rates is a very important but often challenging task, particularly in cases in which data from different datasets are merged into a single database. The false-positive glycosites can be grouped into two sources. The first source is the data acquisition including isolation of nonspecific glycopeptides and analyses of the extracted peptides by MS. The glycosites in this group contain peptides that are correctly identified by SEQUEST search. Because *N*-linked glycosylation occurs in sequences containing the N-X-S/T motif, we filtered the identified peptides with this consensus glycosylation motif to reduce the false-positive peptides. The second source of false-positive glycosites is from peptides that are incorrectly identified by SEQEST search. In the present analysis, the false-positive error rate from SEQUEST search was estimated by the PeptideProphet statistical model. One significant advantage of establishing the automated infrastructure in this work is that computed peptide probabilities from PeptideProphet allow estimation of the likelihood of correct identification of each identified glycosite.

**Figure 3:**
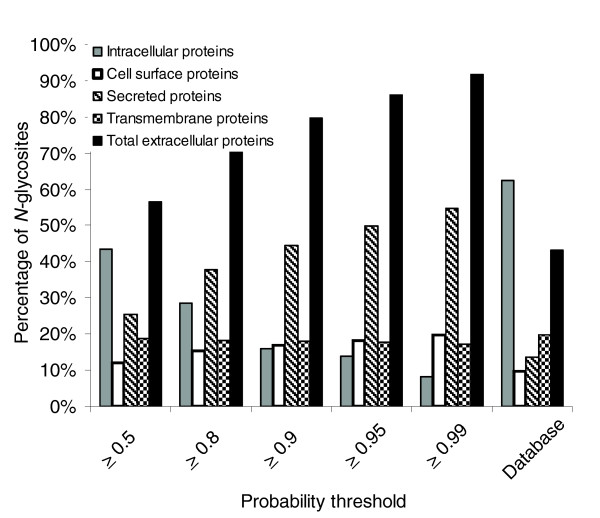
Ratio of identified *N*-linked glycosites identified from proteins predicted as intracellular proteins and extracellular proteins. The extracellular proteins include secreted proteins, cell surface proteins, and transmembrane proteins. The findings are expressed a function of probability stringency.

To assess the overall false-positive rate of identified *N*-linked glycosites using a particular probability threshold on the number of identified *N*-linked glycosites, we filtered the identified *N*-linked glycosites using PeptideProphet probability thresholds *P *≥ 0.5, 0.8, 0.9, 0.95 and 0.99. Because protein glycosylation, in particular *N*-linked glycosylation, occurs in proteins destined for extracellular environments [25], we also calculated the fraction of *N*-linked glycosites that are derived from proteins predicted as 'intracellular proteins' or 'extracellular proteins'. Decreasing the probability threshold increases the number of unique *N*-linked glycosites identified as well as the false-positive rate estimated by the rate of incorrect assignment of *N*-linked glycosites to intracellular proteins. Table 3 indicates the number of unique *N*-linked glycosites derived from intracellular and extracellular proteins (including secreted proteins, cell surface proteins, and transmembrane proteins) as a function of the PeptideProphet probability values. As expected, we observed that the percentage of unique *N*-linked glycosites derived from intracellular proteins decreased while extracellular proteins increased with increasing stringency of the identification criteria (Figure 3). At the highest peptide probability score of 0.99 from SEQUEST search, 8% of the identified *N*-linked glycosites were from intracellular proteins (Figure 3). For comparison, of the 52,442 unique N-X-T/S motif containing potential *N*-linked glycosites from human protein database, 32,770 unique N-X-T/S *N*-linked glycosites are predicted to come from intracellular proteins, representing 62.5% of the total N-X-T/S motif containing sites (Tables 1 and 3, and Figure 3). This indicates that our glycopeptide capture method has significantly enriched the extracellular proteins, and the fraction of glycosites from intracellular proteins is a reasonable estimation of the overall false-positive rate that can result from peptide assignment from SEQUEST search, nonspecific glycopeptide isolation, and peptide analysis using MS/MS.

The most stringent threshold of *P *≥ 0.99 produced 1522 unique *N*-linked glycosites, of which 8% of *N*-linked glycosites were assigned to proteins predicted as being intracellular proteins. Because a 0.99 probability threshold has a very low false-positive error rate (with <1% error rate for peptide assignment), we assumed that at least some of the proteins not annotated as 'extracellular proteins' might represent mis-prediction in the protein subcellular localization. Indeed, closer examination of the data showed that at least some of the identified *N*-linked glycosites were from proteins that were known to be extracellular proteins (carboxypeptidase N 83 kDa chain, and different isoforms of immunoglobulins) but incorrectly annotated as intracellular proteins. Therefore, the real error rate might be lower than the error rate estimated from the percentage of intracellular proteins.

Using a probability score of *P *≥ 0.99 as cutoff, UniPep is currently populated with 1522 identified *N*-linked glycosites. As discussed above, because at this stringency a fraction of the true positive glycosites are lost, we provide on the UniPep website the option for users to browse the *N*-linked glycosites generated at the lower *P *thresholds at the user's own judgment (subject to P ≥ 0.5). Using probability thresholds with lower false-negative rates will be useful in those instances in which a larger number of potential target peptides needs to be identified (Tables 2 and 3).

### Experimental identification of *N*-linked glycosites

To determine which of the potential *N*-linked glycosites were actually glycosylated and can be experimentally confirmed in a variety of samples, we isolated and analyzed *N*-linked glycosites from plasma, cerebrospinal fluid (CSF), and various tissue and cell sources using solid-phase extraction and MS/MS [17]. The resulting spectra were processed through the informatics system described above and entered into UniPep. Currently, the database contains data generated in three different laboratories.

The deglycosylated peptides isolated from whole plasma or plasma depleted of six high abundance proteins using the glycopeptide capture method [12,17] were separated by two-dimensional (strong cation exchange chromatography [SCX] followed by reverse phase) liquid chromatography (LC) and analyzed by electrospray ionization (ESI)-MS/MS on LCQ or LTQ ion trap, or quadrupole time-of-flight (qTOF) mass spectrometers. Collectively, these measurements identified 828 *N*-linked glycosites at a minimum probability threshold of 0.99 (Table 4).

Formerly *N*-linked glycopeptides were isolated from CSF using the method developed by Zhang and coworkers [7]. The deglycosylated peptides were divided into two halves. One half was separated by a two-dimensional microcapillary high-performance liquid chromatography (LC) system, which integrated a SCX column with two alternating reverse phase C18 columns, followed by analysis of each peptide with MS/MS in an LCQ ion trap. The other half of the CSF sample was separated using offline reverse phase chromatography and spotted onto a stainless steel MALDI plate for a total of 576 spots per plate. In total, four MALDI plates were spotted and analyzed by a 4700 Proteomic Analyzer (Applied Biosystems, Foster City, CA, USA). A total of 407 unique *N*-linked glycosites at a minimum probability threshold of 0.99 were identified from CSF including 113 unique *N*-linked glycosites that were only identified in CSF (Table 4).

*N*-linked glycosites from cell and tissue extracts were isolated and identified using essentially the same protocols as for plasma proteins, except that for some cell lines (Jurkat, Ramos) the cell surface was labeled with biotinylated hydrazide on the intact cells to achieve high selectivity for cell surface proteins (Wollscheid and coworkers, unpublished data). In addition to the Ramos and Jurkat cells, SK-BR-3 breast cancer cells, LNCaP prostate cancer cells, primary bladder and prostate cancer tissue, and a primary liver metastasis of prostate cancer were processed by homogenizing tissues or cells followed by solid phase extraction of glycopeptides [17]. The data from each tissue or cell line are summarized in Table 4 and the sequence of the peptides identified from the respective sources is contained in the UniPep database.

After searching the human IPI sequence database with the whole dataset and statistical filtering of the resulting search, the results collectively identified 1522 unique *N*-linked glycosites, maximally representing 1391 proteins at a PeptideProphet score of ≥0.99 (Table 4); 447 proteins were identified by at least one unique *N*-linked glycosite that represents just a single protein in the database.

We also used the number of redundant observations of the same peptide in the dataset as a crude estimate of the corresponding protein's abundance. Similar to gene expression profiling, in which the abundance of a particular transcript can be estimated from the number of observations of a specific expressed sequence tag (EST) counts [44], the number of spectra acquired in a specific body fluid, cell type, or tissue type representing a particular peptide can be used to estimate the relative protein abundance [45]. A total of 173,841 spectra were used to identify the *N*-linked glycosites with PeptideProphet score at least 0.99 in the UniPep database (Table 4). As expected, we observed a wide range identification frequency assigned to a specific *N*-linked glycosite in plasma (from as high as 13,797 spectra assigned to a single *N*-linked glycosite to only a single spectrum used to assign a *N*-linked glycosite; 10^4 ^dynamic range). The highly abundant plasma proteins generated the *N*-linked glycosites (MVSHHN#LTTGATLINEQWLLTTAK, and NLFLN#HSEN#ATAK) from haptoglobin and (ADTHDEILEGLNFN#LTEIPEAQIHEGFQELLR and YLGN#ATAIFFLPDEGK) from α_1_-antitrypsin, which represented more than 20% of the total collision-induced dissociation spectra used for positive peptide identification. In contrast to the *N*-linked glycosites identified from plasma, cells, and tissues have narrower dynamic range of protein abundance.

**Figure 4:**
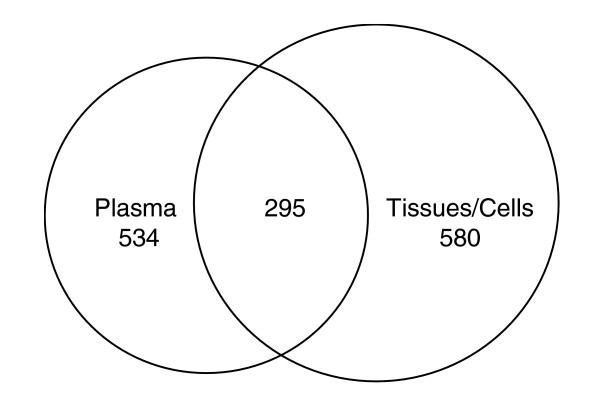
Comparison of number of *N*-linked glycosites commonly or uniquely detected from plasma and tissues/cells. Shows the overlap of *N*-linked glycosites identified in plasma with tissues or cells.

Most cell surface proteins or secreted proteins from cells or tissues are glycosylated. Therefore, if they are secreted or otherwise released into the bloodstream, then they should be observable from plasma using selective *N*-linked glycosite isolation and MS. Such proteins detected and quantified in plasma should be highly informative sentinels reporting the state of the tissue of their origin. We therefore tested the extent to which *N*-linked glycosites observed in cells or tissues could also be detected in plasma. The results show that 295 *N*-linked glycosites are commonly identified from tissues/cells and plasma (Figure 4). This indicates that proteins from tissues or cells are also detectable in plasma, suggesting that *N*-linked glycosite patterns in plasma could potentially be used to detect the status of tissues in the human body remotely.

In the present study, we established a database of *N*-linked glycosites, an informatics pipeline to populate the database with data of consistent quality, and generated an initial dataset of *N*-linked glycosites covering minimally 3% of the possible human *N*-linked glycosites. This database will serve as a resource for glycobiology. In addition, because the majority of currently known cancer biomarkers are known to be glycosylated [46], the database will also significantly contribute to the development of fast, sensitive, robust, and portable mass spectrometric assays to identify and quantify candidate biomarkers [19]. The accurate mass and time tag approach is such an approach [47] that substantially benefits from a mapped out proteomic space. Because this and other similar strategies transform proteomic analyses from a traditional data-dependant discovery phase into a validation and scoring phase by directly focusing on biologically relevant peptides/proteins, they circumvent some of the difficult issues associated with current methods.

## Materials and methods

### Materials and reagents

For chromatography procedures, we used high performance LC grade reagents purchased from Fisher Scientific (Pittsburgh, PA, USA). PNGase F was purchased from New England Biolabs (Beverly, MA, USA) and hydrazide resin was from Bio-Rad (Hercules, CA, USA). All other chemicals used in this study were purchased from Sigma (St. Louis, MO, USA).

### Purification and fractionation of formerly *N*-linked glycosites from plasma

Four datasets were used to generate *N*-linked glycosites from plasma and the *N*-linked glycopeptides were isolated from plasmausing the method described previously [17]. One set of data was generated at the Institute for Systems Biology (Seattle, WA, USA) using serum or plasma samples from individuals following approval from the Human Subject Institutional Review Board of the Institute for Systems Biology [29]. The second set of data was generated at the Institute for Systems Biology using plasma samples from the HUPO study [30]. The third set of data was generated at the Institute for Systems Biology from serum purchased from Sigma, and the forth set of data was generated by the Biological Systems Analysis and Mass Spectrometry group at Pacific Northwest National Laboratory (PNNL; Richland, WA, USA) [12].

### Purification of glycopeptides from human cerebrospinal fluid

The Human Subject Institutional Review Board of the University of Washington approved the study. All 20 participants, aged 35-45 with a male:female ratio of 1:1, were compensated community volunteers in good health. Once written informed consent had been obtained, CSF samples were collected using a procedure described previously [48,49].

Glycopeptides were isolated from CSF using the method developed by Zhang and coworkers [17] with minor modifications. Briefly, triplicate of 2 ml CSF from pooled CSF samples was processed through glycopeptide capture procedure, and the PNGase F released formerly *N*-linked glycopeptides were collected and dried down in a speedVac (Thermo Electron Corporation, Waltham, MA, USA).

### Purification and fractionation of formerly *N*-linked glycosylated peptides from cells and tissues

Human tissue specimens were obtained from organs surgically removed because of cancer under a human subject approval for prostate and bladder cancer biomarker discovery project supported by the Early Detection Research Network from the National Cancer Institute. Isolation of *N*-linked glycopeptides from tissues was performed with cell free supernatant of collagenase-digested prostate, bladder, and liver metastasis tissues using a procedure described previously [17,50].

Isolation of *N*-linked glycopeptides from cultured SK-BR-3 breast cancer cells used homogenized and fractionated cell lysates and serum-free culture medium. On reaching confluence, the SK-BR-3 cells were rinsed five times with serum-free McCoy's 5a medium to wash out the bovine serum proteins, followed by incubation in serum-free McCoy's 5a medium at 37°C for another 24 hours. Then the conditioned medium fraction was collected and the cells were harvested. Cells were homogenized in 0.32 mol/l sucrose and 100 mmol/l sodium phosphate buffer (pH 7.5), and separated into other three fractions via sequential centrifugations (1000 *g *pellet, 17,000 *g *pellet, and 17,000 *g *supernatant). An aliquot of 1 mg protein from each of four fractions was used for *N*-linked glycopeptide isolation using the procedure described previously [17].

Isolation of *N*-linked glycopeptides from the plasma membranes of lymphocytes was via a modification to the *N*-linked glycopeptide capture method for specific labeling of plasma membrane proteins (unpublished data).

### Analysis of peptides by mass spectrometry

Offline fractionated of peptides isolated from plasma samples by SCX before analysis of each fraction with reverse-phase LC and MS/MS was described previously [41]. Analysis of peptides from CSF samples using integrated SCX and reverse-phase C18 columns was done with a previously described procedure [48,49]. All peptides from other sources were analyzed by online reverse-phase LC followed by MS/MS without further fractionation.

Fractionated peptides were analyzed using different mass spectrometers including LCQ and LTQ mass spectrometers (Finnigan, San Jose, CA, USA) [7,48,49] and the ESI-qTOF mass spectrometer (Waters, Milford, MA, USA), in accordance with the manufacturer's instructions [18].

All acquired MS/MS spectra were searched against the IPI human protein database (version 2.28) using SEQUEST software [51] and processed through the pipeline of tools developed at the Institute for Systems Biology to ensure a consistent and high-quality set of peptide identifications with known probability for each peptide sequence assignment. The database sequence tool was set to the following modifications: carboxymethylated cysteines, oxidized methionines, and an enzyme-catalyzed conversion of Asn to Asp at the site of carbohydrate attachment. No other constraints were included in the SEQUEST searches.

Database search results were statistically analyzed using PeptideProphet, which effectively computes a probability for the likelihood of each identification being correct (on a scale from 0 to 1) in a data-dependent fashion [39]. A minimum PeptideProphet probability score filter of 0.5 was used to remove low probability peptides. The resulted peptide sequences were processed through UniPep database pipeline to map individual N-X-S/T sequon containing peptides to UniPep database (Figure 2).

### Subcellular localization of identified proteins

Signal peptides were predicted using SignalP 2.0 [26]. Transmembrane regions were predicted using TMHMM (version 2.0) [27]. The TMHMM program predicts protein topology and the number of transmembrane helices. Information from SignalP and TMHMM were combined to separate proteins into the following categories: cell surface (proteins that contained predicted noncleavable signal peptides and no predicted transmembrane segments); secreted (proteins that contained predicted cleavable signal peptides and no predicted transmembrane segments); transmembrane (proteins that contained predicted transmembrane segments and extracellular loops and intracellular loops); and intracellular (proteins that contained neither predicted signal peptides nor predicted transmembrane regions). All protein sequences were taken from IPI version 2.28.

### UniPep to interrogate proteotypic *N*-linked glycopeptides for proteins in database

UniPep is a web interface that allows researchers to query a database for a proteotypic *N*-linked glycopeptide of a specific protein. UniPep contains all proteins in the IPI database (version 2.28) with at least one *N*-linked glycosylation sequon, and it allows users to view all of the predicted and identified *N*-linked glycopeptides from a specific protein. The scripts and data were developed within the SBEAMS framework under the PeptideAtlas branch [29]. For each potential *N*-linked glycoprotein, a user can see the protein annotation, predicted subcellular location, and sequence(s) of predicted and identified glycopeptide(s). The uniqueness of a peptide in the database is also presented as number of hits in the database, and for those peptides that are present in multiple proteins, linkage to other proteins in the database is provided. Any predicted glycopeptides identified experimentally are listed as an identified peptide with a PeptideProphet score [39] to allow researcher to evaluate the confidence of the identification. The sequence of the proteins queried is overlaid with different sequence features such as the *N*-linked glycosites, the predicted and identified peptide sequences, signal peptide, and transmembrane segment(s). This information is provided to allow the user to choose an identified or predicted *N*-linked glycosite for a specific protein of interest.

### Data availability

All *N*-linked glycosites identified from plasma, bladder tissues, breast cancer cells, liver cancer tissues, lymphocytes, prostate cancer cells, prostate tissues, and CSF in this study are available from UniPep (Table 3 and 4).

## Figures and Tables

**Table 1:** Distribution of unique tryptic peptides and tryptic peptides containing the N-X-T/S motif over subcellular classes of proteins in the human protein (IPI) database

	Tryptic peptides^a^		Peptides containing N-X-T/S	
	
	Number of peptides^a^	Number of proteins	Number of peptides^a^	Number of proteins
Intracellular	510,685(68.2%^b^)	26,721(66.6%^c^)	32,770(4.4%^b^)	17,475(43.6%^c^)
Secreted	80,069(10.7%)	3,772(9.4%)	7,195(1.0%^b^)	2,772(6.9%^c^)
Transmembrane	114,282(15.3%)	6,375(15.9%)	10,359(1.4%)	4,645(11.6%)
Cell surface	70,126(9.4%)	3,242(8.1%)	5,138(0.7%)	2,166(5.4%)
All extracellular	264,477(35.5%)	13,389(33.4%)	22,692(3.0%)	9,583(23.9%)
Total protein	749,163(100%)	40,110(100%)	52,442(7.0%)	27,058(67.5%)

The human International Protein Index (IPI) database (version 2.28) contains a total of 40,110 protein entries. ^a^Tryptic peptides are defined as peptide sequences that end with Arg or Lys, are not followed by proline, and fall within the mass range from 500 to 5000 Da. ^b^The percentage represents the fraction of total tryptic peptides from the human database (749,163). ^c^The percentage represents the fraction of total proteins from the human database (40,110).

**Table 2:** False-positive and false-negative rates of peptide identifications in liver tissue predicted by PeptideProphet at different probability thresholds

Probability score cutoff	False-negative rate	False-positive rate
0.99	0.6042	0.0025
0.95	0.4037	0.0099
0.90	0.3297	0.0172
0.80	0.2621	0.0304
0.70	0.2252	0.0437
0.60	0.1964	0.0593
0.50	0.1713	0.0787
0.40	0.1440	0.1091
0.30	0.1262	0.1364
0.20	0.1041	0.1877
0.10	0.0724	0.3010
0.00	0.0000	0.9295

**Table 3:** Number of unique *N*-linked glycosites and percentage of sites from intracellular or extracellular proteins using different peptide probability thresholds

	Probability threshold					Database
		
	≥0.5	≥0.8	≥0.9	≥0.95	≥0.99	
Number of unique *N*-linked glycosites	5202	2870	2265	1895	1522	52442
Number of unique *N*-linked glycosites from intracellular proteins	2207	817	363	264	124	32770
Number of unique *N*-linked glycosites from secreted proteins	1326	1086	1011	946	834	7195
Number of unique *N*-linked glycosites from transmembrane proteins	976	523	408	337	263	10359
Number of unique *N*-linked glycosites from cell surface proteins	633	444	383	348	301	5138
Number of unique *N*-linked glycosites from all extracellular proteins	2935	2053	1802	1631	1398	22692

**Table 4:** Summary of *N*-linked glycosites identified from different sample sources with probability score at least 0.99

Sample source	Number of unique glycosites	Number of source-specific glycosites	Number of spectra used for ID
All	1,522		173,841
Plasma	828	433	156,814
Bladder	145	3	1,121
Breast cancer cells	369	135	2,725
Liver	202	13	964
Lymphocytes	288	156	2,847
Prostate cancer cells	71	4	108
Prostate tissue	354	53	3,804
Cerebrospinal fluid	407	113	5,453
